# The Role of Isolation Methods on a Nanoscale Surface Structure and its Effect on the Size of Exosomes

**DOI:** 10.5772/64148

**Published:** 2016-01-01

**Authors:** JungReem Woo, Shivani Sharma, James Gimzewski

**Affiliations:** 1 Department of Chemistry and Biochemistry, University of California, Los Angeles, USA; 2 California NanoSystems Institute, University of California, Los Angeles, California, USA

**Keywords:** AFM, Exosome, Nanoparticles, Immunoaffinity, Surface Roughness, Vesicles, Isolation Method

## Abstract

Exosomes are ∼100 nanometre diameter vesicles secreted by mammalian cells. These emerging disease biomarkers carry nucleic acids, proteins and lipids specific to the parental cells that secrete them. Exosomes are typically isolated in bulk by ultracentrifugation, filtration or immunoaffinity precipitation for downstream proteomic, genomic, or lipidomic analysis. However, the structural properties and heterogeneity of isolated exosomes at the single vesicle level are not well characterized due to their small size. In this paper, by using high-resolution atomic force microscope imaging, we show the nanoscale morphology and structural heterogeneity in exosomes derived from U87 cells. Quantitative assessment of single exosomes reveals nanoscale variations in morphology, surface roughness and counts isolated by ultracentrifugation (UC) and immunoaffinity (IA) purification. Both methods produce intact globular, 30–120 nm sized vesicles when imaged under fluid and in air. However, IA exosomes had higher surface roughness and bimodal size population compared to UC exosomes. The study highlights the differences in size and surface topography of exosomes purified from a single cell type using different isolation methods.

## 1. Introduction

Cells utilize vesicles for the intercellular signalling, transporting and trafficking of metabolites [1–2]. A more collective term, “extracellular vesicle” (EV), is often used as a synonym for “membrane vesicles”, which includes microvesicles, exosomes, apoptic bodies and other vesicles. In this paper, we use the term “exosomes” due to the characteristic size, morphology and specific surface markers present.

Exosomes, which are 30–120 nm-sized vesicles, are of particular interest for potential disease diagnostic markers. Due to their small size, it was once believed that exosomes were random cell debris, but it is now known that they are actively secreted from cells with specific markers [3–6]. Exosomes carry nucleic acids [7], proteins [8] and lipids [9], and can deliver these diverse components to distant recipient cells and thus hold potential as emerging biomarkers for diseases including cancers [10–12]. Nevertheless, the isolation and characterization of purified exosomes from bodily fluids, tissues, or cells of origin are crucial aspects for any downstream biomarker discovery research.

Many isolation procedures and commercially available kits are currently being actively researched in a bid to optimize the enrichment and isolation of purified exosomes in bulk [13]. For the isolation of exosomes, conventional methods utilize sequential centrifugation and ultracentrifugation (UC) to spin down exosomes in cell culture media or bodily fluids [14]. Sucrose density gradient ultracentrifugation is required for additional purification and to minimize protein contamination [15]. Size exclusion chromatography is also employed to purify exosomes based on their physical dimensions [16]. In addition, antibodies targeting exosome surface markers such as CD9, CD63, CD81 and EpCAM are attached to magnetic beads (immunoaffinity; IA) to capture and purify exosomes [15, 17]. After isolation, the purified exosome samples are characterized using various imaging and biochemical techniques. It is difficult to characterize the 3D structure of single exosomes due to their nanometric dimensions. The particle size distribution of extracellular vesicles has been analysed with transmission electron microscopy (TEM), flow cytometry, resistive pulse sensing and nanoparticle tracking analysis [18]. TEM provides standard size distribution information, but suffers from fixation artefacts and non-biocompatible conditions such as low temperature and high vacuum. Flow cytometry is not optimal for exosomes, due to its poor resolution when particle diameters are less than 100 nm. Resistive pulse sensing and nanoparticle tracking analysis resolves particles with diameters ranging from 10–1000 nm, but is limited in terms of resolving heterogeneous vesicle populations. The first structural characterization of exosomes was effected using TEM with immunogold nanoparticle labelling [19]. Based on EM images, exosomes were thought to have ‘cup-shaped structures’. However, atomic force microscope (AFM) and field emission scanning electron microscope images subsequently revealed that exosomes are globular vesicles, similar to other vesicles present in cells [20].

One of the limitations of these bulk isolation methods is that they preclude identification of heterogeneity among exosome sub-populations. It is known that exosomes secreted by the same originating tissues, cell types and single cells, may vary in terms of composition, size and density [21], which can have significant implications for studies in exosome biology and their role as disease biomarkers in various pathologies [22]. The commonly used NanoSight tracking analysis (NTA) is a comparable non-microscopic method for measuring exosome sizes and distributions. Many researchers have utilized and compared both microscopic and non-microscopic methods for obtaining exosome sizes [23]. However, NTA does not provide quantitative surface information at sub-nm resolutions. Recently, we reported high-resolution images of glioblastoma exosomes using PeakForce AFM imaging and showed that their nanofilament structure may influence the delivery of exosomes into recipient cells [24]. Here, using high-resolution AFM imaging, we investigate the nanoscale morphology and structural heterogeneity in exosomes isolated from glioblastoma U87 cell lines, and the influence of particular isolation methods. Quantitative assessment of exosomes at the single vesicle level reveal nanoscale variations in morphology, surface roughness and counts of exosomes isolated using two methods, i.e., ultracentrifugation (UC) and immunoaffinity (IA) bead purification with widely used exosomal markers (CD9, CD63, CD81 and EpCAM). Our AFM imaging data on single vesicles reveals differences in the size and surface topography of exosomes purified from the same cell type but using different isolation methods. We attribute the observed variations in size and surface roughness of isolated exosomes to the selection and/or reassembly of receptors on the surface of isolated exosomes.

## 2. Results

### 2.1 Single exosome images show differences in structure depending on purification method

**Figure 1.**
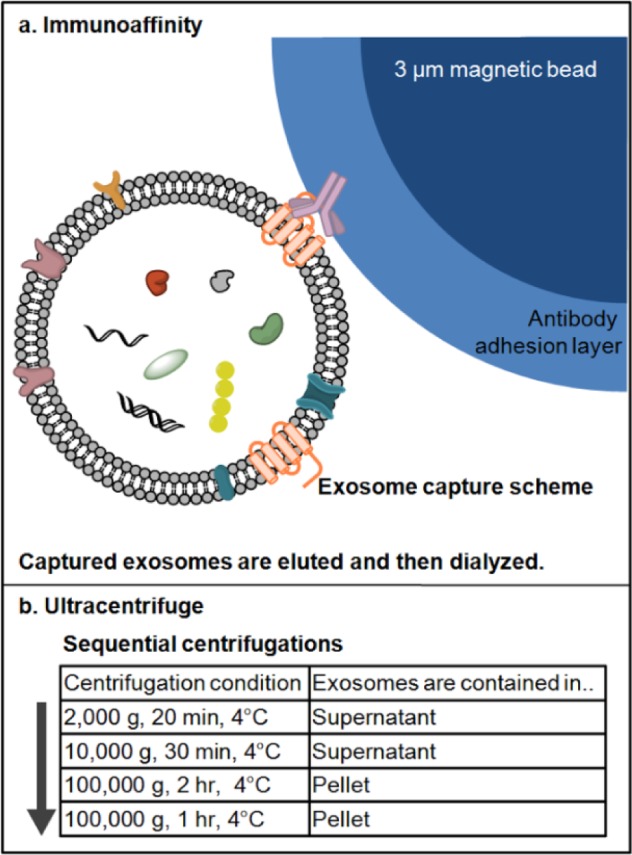
(a) The scheme of exosome isolation by immunoaffinity magnetic beads kit method. For capturing exosomes, antibodies against four different surface markers (CD9, CD63, CD81 and EpCAM) were immobilized on the surface of 3 μm magnetic beads. Each antibody was modified separately on the beads and the mixture of the antibody-beads used to capture antibodies from cell culture media (not drawn in scale). After capture and elution, isolated exosomes were diluted and dialyzed against PBS prior to characterization/storage; (b) flowchart of exosome isolation by ultracentrifugation method. The exosome-containing media was centrifuged sequentially to remove particles denser than exosomes, then centrifuged to pellet down exosomes (exosome buoyant density of 1.10–1.24 g/mL). The flowchart indicates each centrifugation step and the location of exosomes. Exosomes were pelleted following ultracentrifugation at 100 000 g and the pellet was washed with PBS, then ultra centrifuged again. The final exosome pellet was re-suspended in either deionized water or PBS and characterized/stored.

We investigated the structural differences among isolated exosomes at the single vesicle level between those purified using two different methods, i.e., UC and IA (Fig. 1). The antibodies on the magnetic beads were against four different antigens: CD9, CD63, CD81 and EpCAM. Each antibody is immobilized on separate bead solutions; then, the individual antibody-beads are mixed together. Since the antibodies select exosomes according to their surface proteins, it is possible that exosomes with specific surface markers may result in the size selection of the entire exosome population. On the other hand, ultracentrifuge utilizes the density difference in the sample to purify exosomes. Ultracentrifugation [14] is the most commonly used method for concentrating exosomes that include differential centrifugation steps up to 100 000 × *g*. Though the method is time consuming (4–5 hours), requires an ultracentrifuge and is inefficient with regard to exosome yield (5–25% recovery), it nevertheless enables label-free isolation of exosomes.

#### 2.1.1 The structural characterization of native exosomes in fluid: Peak force imaging

**Figure 2.**
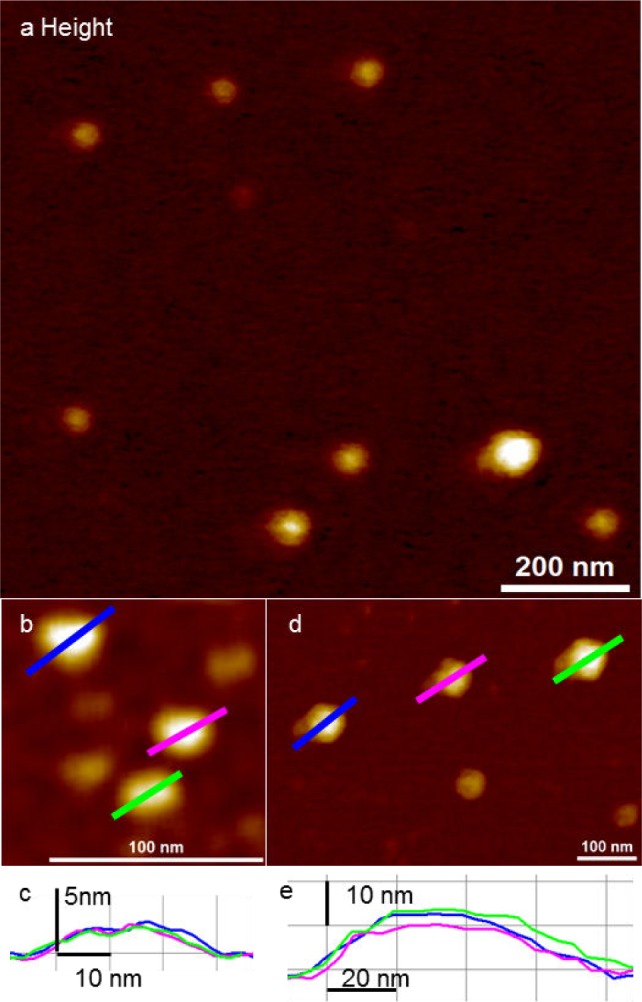
Detection of IA exosomes by peak force AFM imaging in fluid; (a) representative topographic image of exosomes showing varying vesicle size; (b) close-up of smaller IA exosomes. The smaller IA exosomes were 22.5 ± 3.5 nm in diameter and 1.8 ± 0.3 nm in height; (c) cross-section of (b) shows IA exosome dimensions to be approximately 30 nm in diameter and with a height of 2.3 nm; (d) close-up of larger IA exosomes. The dimensions of bigger IA exosomes are a diameter of 64.6 ± 9.2 nm and a height of 8.1 ± 3.1 nm; (e) the cross-section of (d) show IA exosome dimensions of approximately 80 nm in diameter and a height of 12 nm.

Peak force mode is a recently developed AFM imaging mode enabling very low imaging forces (less than 1 nN) for the detection of exosomes in fluid. Figure 2 (a) shows IA exosomes imaged using AFM in fluid. IA exosomes show globular structures of varying sizes with an average diameter of 39 ± 22 nm in width and an average height of 4.3 ± 3.7 nm. Figure 2 (b) shows a close-up image of the smaller IA exosomes and cross-sections of the exosomes in Figure 2 (c). The smaller IA exosomes have a diameter of ∼30 nm and a height of ∼2.5 nm. On the other hand, the bigger IA exosomes are shown in Figure 2 (d) and their cross-sections are displayed in Figure 2 (e). The bigger IA exosomes have a diameter of ∼80 nm and a height of ∼10–12 nm. Both cross-sections from smaller and bigger exosomes confirm their globular shapes, as expected under native conditions. When compared to air AFM imaging, the size distributions remain similar.

**Figure 3.**
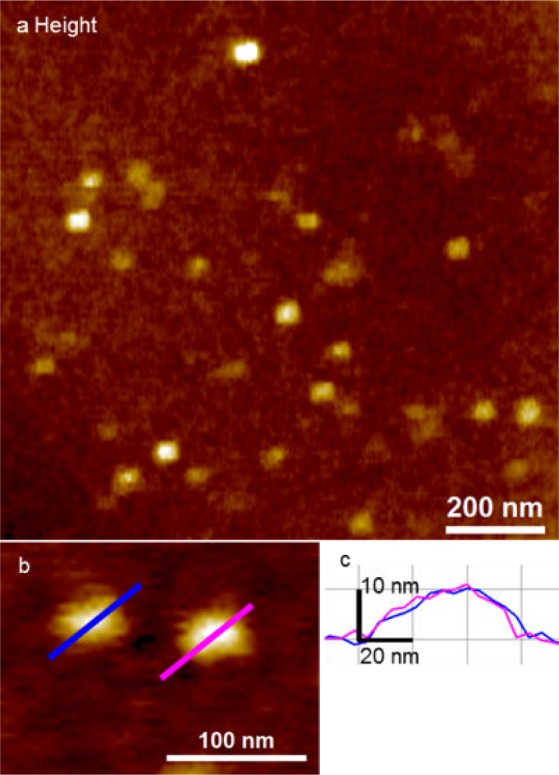
Detection of UC exosomes by peak force AFM imaging in fluid; (a) representative topographic image of UC exosomes. UC exosomes showed a single Gaussian population in fluid imaging; (b) close-up of UC exosomes showing a diameter of 49 ± 9 nm and a height of 7.8 ± 2.8 nm; (c) the cross-section of (b) shows UC exosome dimensions of approximately 60 nm in diameter and with a height of 10 nm.

UC exosomes also show a globular shape in AFM images in fluid (Figure 3 (a)). UC exosomes showed a single population with an average diameter of 49 ± 9 nm and an average height of 7.8 ± 2.8 nm. A smaller scan area of the UC exosomes are shown in Figure 3 (b) and their cross-sections are shown in Figure 3 (c). The UC exosomes have a diameter of ∼60 nm and a height of ∼10 nm.

#### 2.1.2 The structural characterization of native exosomes in air: Tapping mode imaging

**Figure 4.**
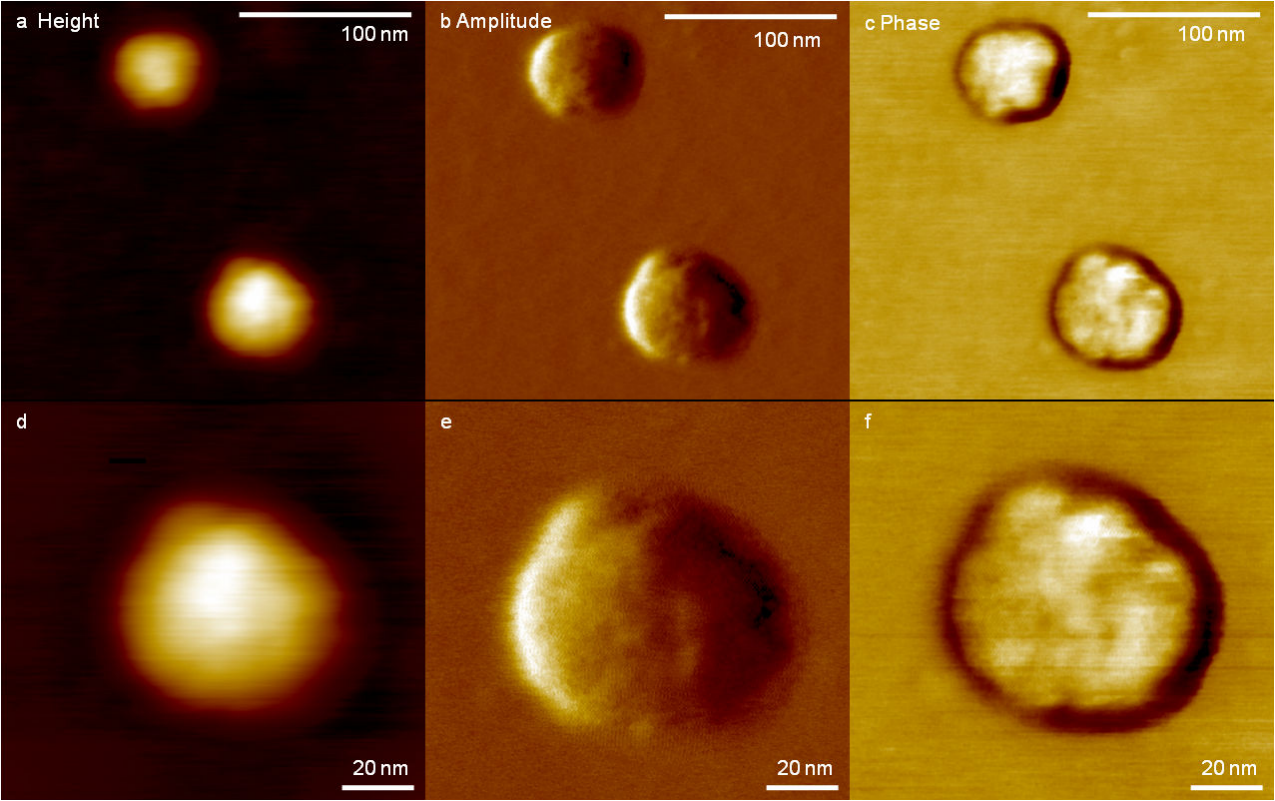
High-resolution images of IA exosomes obtained by AFM imaging in air; (a) representative topography, (b) amplitude and (c) phase images of exosomes shown while the boxed exosome is magnified in (d) topography, (e) amplitude and (f) phase images. The diameter and height of the exosome in (d) is 76 nm and 9.3 nm, respectively. The phase channel shows the rough surface of the exosome.

Exosomes were isolated using the IA method and their nanoscale structure was obtained using tapping mode AFM imaging in air. Figure 4 (a)–(c) shows the detailed structure of single exosomes isolated by the IA method. Single exosomes show a globular structure with a diameter of 76 nm and a height of 9.3 nm. The dimensions of the exosomes are similar to the values previously reported [21]. The detailed structure of a single exosome is shown in Figure 4 (d)–(e). Surface roughness of the exosomes within a 40 nm × 40 nm area, shown as a box in Figure 4 (d), were measured as Ra (arithmetic average of the absolute values, 1.48 nm) and Rq (root mean squared, 1.17 nm). The roughness of the exosome surface is clearly evident in the phase channel in Figure 4 (f). The phase image is determined by the lag between incident resonant oscillations and the output signal oscillations, which are sensitive to surface properties such as elasticity, adhesion and friction.

### 2.2 Exosomes show different size distributions depending on purification methods

**Figure 5.**
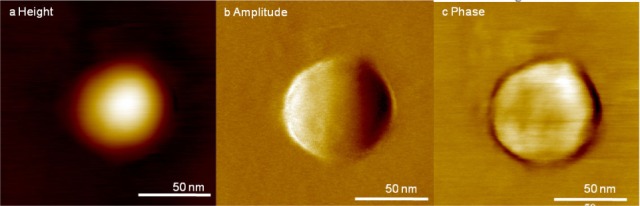
High-resolution images of UC exosomes obtained by AFM imaging in air; (a) representative topography, (b) amplitude and (c) phase images of exosomes. Inset (a) is the cross-section of the exosome for the height channel. The dimensions of the exosome in (a) are a diameter of 79 nm and a height of 6.2 nm. The phase channel shows the smooth surface of the exosome.

**Figure 6.**
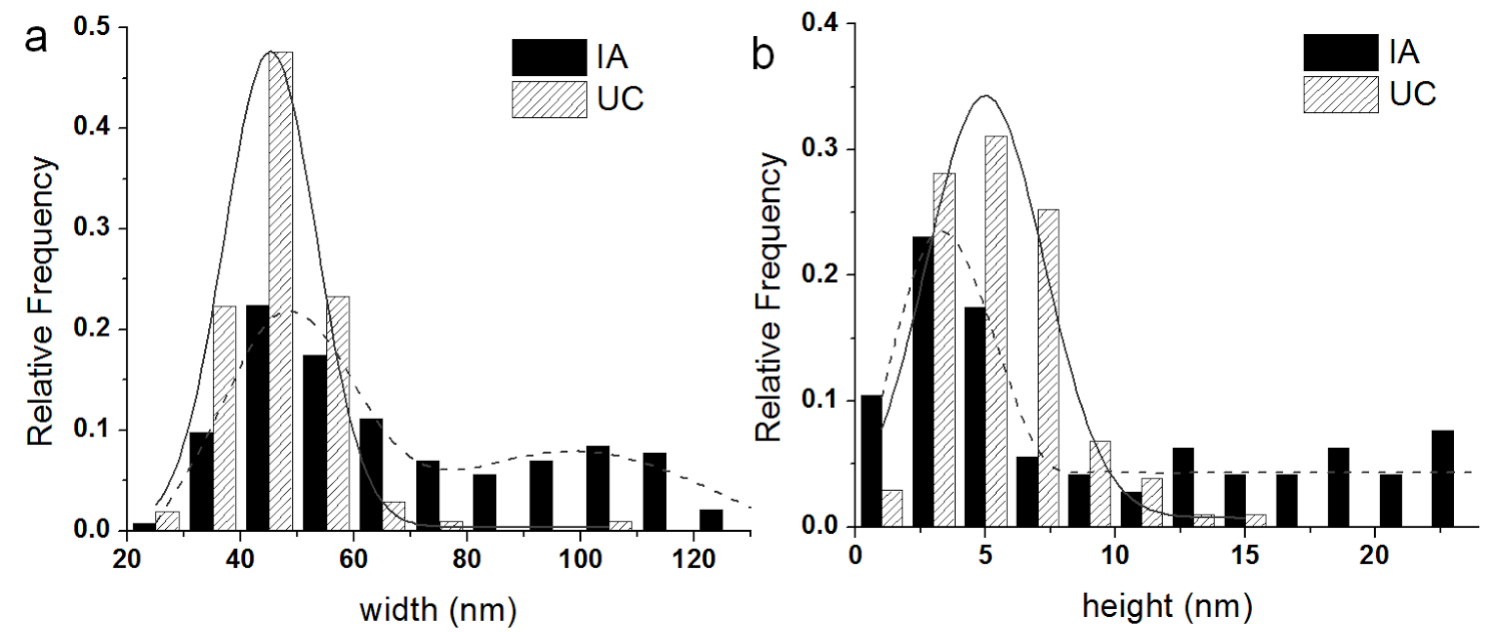
The size distributions of exosomes depending on isolation methods. The width distributions (a) and the height distributions (b) are shown with Gaussian fits. The width distribution of exosomes isolated by IA show a bimodal population, with the first peak at 23.9 ± 3.2 nm and the second peak at 54 ± 18 nm. The width distribution of exosomes isolated by UC show a single Gaussian population with the peak at 45.2 ± 0.1 nm. The height distribution of exosomes show a single Gaussian population for both methods, with the peak at 3.2 ± 0.5 nm for IA and 5.0 ± 0.3 nm for UC. The width and the height of exosomes from IA and UC were significantly different at p<0.05.

We measured the dimensions of IA and UC (Fig. 5) exosomes to determine if their size distributions depended on the purification method. The diameter and height were measured for more than 100 exosomes. The average diameter was 68.1 ± 26.5 nm and 45.9 ± 10.3 nm for IA and UC, respectively. The average height was 9.6 ± 7.7 nm and 5.5 ± 2.4 nm for IA and UC, respectively. In the average values, the dimensions of the exosomes seemed to be similar, while IA exosomes were ∼30% bigger than UC exosomes. Figure 6 shows a histogram of (a) the diameter and (b) the height of IA and UC exosomes, which are markedly different. While the UC exosomes show normal distribution (solid lines) in diameters and heights, the IA exosomes display bimodal distribution (dashed line). The IA exosomes show wider bimodal distributions. The first peak was observed at 23.9 ± 3.2 nm in width and 3.2 ± 0.5 nm in height, and the second peak at 54 ± 18 nm in width. In the case of UC exosomes, it shows relatively narrow singular distributions with a peak at 45.2 ± 0.1 nm in width and 3.2 ± 0.5 nm in height. The difference in size distribution may stem from the isolation mechanism employed. For the UC method, exosomes were isolated based on buoyant density and likelihood of resulting in a single Gaussian population. However, the IA method selects exosomes based on surface antigens. As an IA kit contains four different antibodies, during purification, exosomes are affinity-purified based on surface markers, which may result in wider bimodal size distributions.

## 3. Discussion

Exosomes have the potential to be utilized as disease biomarkers that carry signature biomolecules (nucleic acids, proteins and lipids) from the secreting cells of origin [7, 9, 25–29]. However, due to their sub-100 nm size and low abundance (∼10^6–9^ vesicles per ml) in extracellular environments, it is challenging to isolate and characterize single exosomes. During a special workshop presented by the International Society for Extracellular Vesicles (ISEV), researchers and experts discussed the standardization of exosome isolation, handling and characterization. Although a consensus was not reached [13], Lötvall et al. later proposed basic experimental requirements for defining exosomes [30]. The proteomic and genomic contents of exosomes, depending on the isolation method, have since been compared and evaluated [15, 26, 31–33]. In addition, evaluation of the structural integrity and morphology of isolated exosomes is important for standardization and downstream biomolecular analysis. In this paper we focus on single exosome characterization using ultracentrifugation and immunoaffinity capture to elucidate their nanoscale structure on an individual basis. This was achieved using high-resolution AFM imaging of U87 cell-derived exosomes. We discovered that quantitative assessment of exosomes at the single vesicle level reveals nanoscale variations in the morphology, surface roughness and counts of exosomes isolated, which differ depending on the extraction method used – ultracentrifugation (UC) or immunoaffinity (IA) bead purification using exosomal markers (CD9, CD63, CD81 and EpCAM). Our study is the first to reveal the high-resolution structural variations (morphology and size) and surface inhomogeneity (topographic roughness) of exosome populations at the single vesicle level.

Our results indicate distinct differences between UC and IA exosomes purified at the single exosome level. Although both methods yield globular vesicles, we found that UC exosomes have smooth surface, whereas IA exosomes exhibit a distinct roughness. Secondly, the size distributions were different. UC exosomes showed a single Gaussian distribution, whereas IA exosomes showed a broader bimodal distribution. Furthermore, peaks in IA exosome size histograms clearly indicate that IA exosomes are either smaller or larger than UC exosomes. This was observed under both AFM imaging techniques and environments, i.e., tapping mode in air and peak force mode in fluid. We attribute the measured variations to the specific isolation protocol. Table S 1 summarizes the differences in two exosome isolation methods, showing a side-by-side comparison and the pros and cons of each method, including the experimental details such as time, yield, sample starting/final volumes and equipment used. On the one hand, UC relies on the size and density (1.12–1.19 g/ml) of the vesicles to precipitate, at high centrifugal forces, intact unlabelled exosomes from the solution (Figure 3 and 5). On the other hand, IA-isolated exosomes result from a combination of the selective sorting of vesicles enriched in either of the four targeted exosomal markers, varying in binding affinities, as well as the process of exosome elution from magnetic beads. Affinity purification involves specific non-covalent binding interactions between immobilized target exosomal marker antibodies (CD9, CD63, CD81 and EpCAM) on beads with ligands on the exosome surface. Addition of the cell culture supernatant (a complex mixture containing exosomes) allows exosomes to bind according to their specific affinity to the immobilized antibody molecules. However, to elute exosomes from the beads, it is necessary to break the antigen-antibody binding. The binding force differs for each specific antibody-antigen, ranging from 50–500 pN [34–35] compared to the covalent binding force ranges of 1–2 nN [36–38]. Although appropriate buffer conditions for binding in affinity purification vary, antibody-antigen binding is generally most efficient in aqueous buffers at a physiological pH and ionic strength, such as phosphate-buffered saline (PBS), which was employed in our protocol. After washing away the non-bound components of the complex mixture, the captured exosomes are released and recovered (i.e., eluted) from the “bead-immobilized antibody” using buffer conditions that disrupt the affinity interaction. Methods such as raising or lowering the pH, adding a mildly active detergent, or altering the ionization state are commonly used. The proprietary exosome elution buffer (Exocap, JSR Life Science) enabled the recovery of intact exosomes, as confirmed by AFM images (Figure 2 and 4).

**Figure 7.**
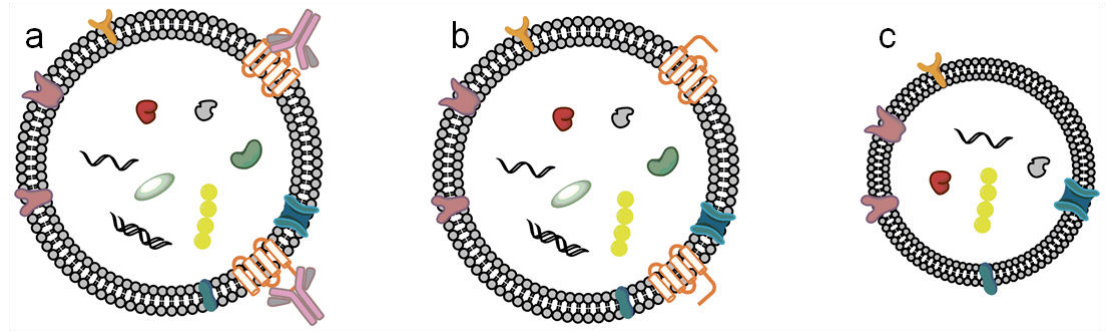
Three possible IA exosomes eluted from beads; (a) antigen-antibody interaction may remain intact, while the antibody-bead linkers are disrupted during elution. Thus, antibodies are attached on the eluted IA exosomes, resulting in expanded sizes; (b) ideally, only the antigen-antibody interaction is affected and eluted IA exosomes will be the same as UC exosomes; (c) tetraspanins (CD9, CD63 and CD 81) are pulled out during the elution, causing IA exosomes' lost volume and partial contents.

There are some possibilities that can explain the observed differences in exosome distribution and surface structure at the single vesicle level. First, the surfactant on the magnetic bead may transfer to the exosome surface. While this might explain the increase in roughness and size of the exosome, it cannot explain their shrinkage (width and height) as observed in the bimodal distribution (see Figure 6). Another possibility is that the IA exosomes are altered during the elution step (Figure 7). When exosome-bead complexes are incubated with an elution buffer, not only is antigen-antibody interaction affected, but also the exosome itself, as well as antibody-bead linkers. To minimize such unwarranted effects, the elution buffer was diluted and immediately dialyzed against PBS. However, the antibody-bead linker may nonetheless be disrupted and few antibodies will remain on the exosome resulting in the increased apparent size of the extracted exosome (Figure 7(a)). Additionally, it is possible for tetraspanins to be extracted from the exosome surface, resulting in the reassembly of exosomes by the active detergents in the elution buffer, which will lead to the underestimation of exosome dimensions (Figure 7 (c)). Furthermore, UC-isolated exosomes showed a smoother surface (Ra; 1.16 nm and Rq; 0.95 nm, see also Figure 5 (c)) and narrower size distributions. In contrast, in the case of IA exosomes, we observed a wider bimodal distribution of dimensions both in diameter and height (Figure 6), and accompanied by a higher surface roughness compared to UC exosomes (Ra; 1.48 nm and Rq; 1.17 nm, see also Figure 4 (c), (f)). All of these observed differences are consistent with our interpretation that using the IA method results in subtle changes in the exosome surface layer.

In summary, our AFM images of IA- and UC-isolated exosomes confirm in both cases high stability, nonaggregation and vesicle integrity, suggesting the retention of bio-functionality and suitability for downstream analysis, which is required for thorough investigation in future functional, microRNA and/or protein assays. Exosomes are heterogeneous vesicles with various surface markers [39]. Since exosomes show sub-populations with different markers and sizes, it is desirable to perform more analysis on single vesicle levels than ensemble qualifications. In the past few years, nanotechnology-based isolation techniques [40] have been developed for isolating exosomes. Such advances make it further evident that detailed characterizations of exosomes are essential in order to advance a biological understanding and the biotechnological exploitation of exosomes, at the single vesicle and sub-vesicular level [41–42]. Although the limitations of current technologies do not support the sorting of exosomes according to sizes, it nonetheless provides insight about the analysis of exosome ensemble data when sub-populations are acknowledged.

Both the UC and IA methods produce intact globular exosomes at the single vesicle level. The advantage of the IA method is speed and ease of use. In the current study, a mixture of four capture antibodies (anti-CD9, CD63, CD81 and EpCAM) was used for IA purification; hence, we cannot exclude the co-purification of various exosome subpopulations. On the other hand, purification by UC is based on the size and density of exosomes and as such, can be expected to yield a more homogeneous size distribution. In future, evaluating the size distribution of exosomes obtained from single capture antibodies will be additionally advantageous. However, this clearly indicates that the nanoscale characterization of isolated exosomes is advisable, particularly in detailed proteomic investigations such as mass spectroscopy studies, where biological purity and the chemical properties of a sample greatly influence protein identification [43]. In addition, our data presents more implications pertaining to cell biological functions [44], as UC exosomes and IA exosomes display different sub-populations. For example, UC exosomes with smoother topographic surfaces may have distinct physical characteristics for cell binding and activation, whereas some of the epitopes of surface markers on IA exosomes may not be accessible, due to their rough surface. In addition, exosomes isolated from each individual surface marker may have different biochemical, genomic, or proteomic properties and may have the potential for being employed as diagnostic biomarkers.

## 4. Materials and Methods

### 4.1 U87 cell culture

U87 cells were cultured in Dulbecco's Modified Eagle Medium (DMEM) and supplemented with 10% heat inactivated fetal bovine serum (FBS), 100 units/mL Penicillin G, and 100 μg/mL Streptomycin. Cells were incubated at 37°C and 5% carbon dioxide. At approximately 80% confluence, cells were washed with PBS and passaged using a 0.25% trypsin-EDTA treatment for dissociation.

### 4.2 Exosome isolation

U87 cells were cultured in six 60 mm Petri dishes (falcon) with FBS-originated-exosome-free media (as instructed in the protocol by Théry; FBS was ultracentrifuged at 100 000 g for 2 hours at 4°C, then filtered with a 0.22 μm sterile filter) for 48 hours; after 48 hours, the media containing U87 exosomes was isolated. Total cell count was 2×10^7^ and 24 mL of U87-exosome-containing media was obtained. For the following isolation methods, the same batch of media was used.

### 4.3 Isolation of exosomes using immunoaffinity (IA) magnetic beads kit method

The procedure suggested in the manufacturer's manual (JSR Life Science, Tokyo, Japan) was followed; 200 μL of U87 exosome-containing media was incubated with 100 μL of capture beads for 60 min at room temperature (RT) on a shaker. Beads were separated from the supernatant by a magnet and washed with a 0.5mL wash buffer three times; 50 μL of elution buffer was added to the beads and the beads were gently re-suspended, then incubated without mixing for 3 min at RT. Beads were separated and the supernatant was transferred to a Slide-A-Lyzer™ Dialysis Cassette (Thermo Fisher Scientific, MA, USA), then dialyzed against PBS. Purified exosomes were stored at 4°C until AFM imaging.

### 4.4 Isolation of exosomes using the ultracentrifugation (UC) method

Exosome isolation using ultracentrifugation was followed by a previously published protocol (Figure 1) [14]. To remove cells/debris, the exosome-containing media was centrifuged at 2000g for 20 min at 4°C and supernatant 1 was isolated. Then, supernatant 1 was centrifuged at 10 000g for 30 min at 4°C to remove microvesicles and again, supernatant 2 was carefully isolated. To isolate exosomes, supernatant 2 was ultracentrifuged at 100 000g for 2 hours at 4°C and supernatant 3 was discarded. To wash exosomes, the pellet was re-suspended in 1 mL PBS and the mixture was ultracentrifuged at 100 000g for 1 hour at 4°C, and supernatant 4 was discarded. Purified exosomes were re-suspended in 100 μL of PBS and stored at 4°C until AFM imaging.

### 4.5 Sample preparation and air imaging

Exosome samples purified from U87 were incubated on freshly cleaved mica for 5 min, washed with deionized water to remove any unbound exosomes and air-dried overnight. Samples were imaged by Dimension ICON (Bruker Instruments, CA, USA) using the tapping mode via TESP cantilever (Bruker Instruments, CA, USA) and images were recorded at 1024 samples per line at 1 Hz. Image processing was done using SPIP™ software. For exosome yield calculation, images at a size of 1 μm × 1 μm and a resolution of 512 samples per line, at 1 Hz, were used. For exosome surface roughness measurements, images were processed with SPIP™ to obtain Ra (arithmetic average of the absolute values) and Rq (root mean squared) values.

### 4.6 Sample preparation and fluid imaging

To anchor exosomes on the surface, (3-Aminopropyl) triethoxysilane (APTES) modified mica was prepared; 10 μL of 10% APTES solution was incubated with clean, freshly cleaved mica discs in a vacuum chamber overnight. Nitrogen gas was purged and the mica discs were stored in a nitrogen gas chamber; 50 μL of the exosome sample was incubated on an APTES modified mica disc for 10 min. To remove unbound exosomes, mica was washed with deionized water four times; 50 μL of deionized water was added on mica prior to imaging. Samples were imaged using Dimension Icon (Bruker Instruments, CA, USA) with MLCT-E cantilevers (Bruker) for fluid imaging in QNM peak force mode [38, 45]. AFM probes were calibrated using a thermal method and images were taken at 256 samples per line, at 0.6 Hz, then processed by SPIP™.

## Abbreviations

AFM: atomic force microscopy;
TEM: transmission electron microscopy
UC: ultracentrifugation
IA: immunoaffinity
